# Does the RAAS play a role in loss of taste and smell during COVID-19 infections?

**DOI:** 10.1038/s41397-020-00202-8

**Published:** 2020-12-15

**Authors:** Heloise R. Luchiari, Ricardo J. Giordano, Richard L. Sidman, Renata Pasqualini, Wadih Arap

**Affiliations:** 1 Department of Biochemistry, Institute of Chemistry, University of São Paulo, São Paulo, SP Brazil; 2 Department of Neurology, Harvard Medical School, Boston, MA USA; 3 Rutgers Cancer Institute of New Jersey and Division of Cancer Biology, Department of Radiation Oncology, Rutgers New Jersey Medical School, Newark, NJ USA; 4 Rutgers Cancer Institute of New Jersey and Division of Hematology/Oncology, Department of Medicine, Rutgers New Jersey Medical School, Newark, NJ USA

## Introduction

The new Severe Acute Respiratory Syndrome (SARS) coronavirus, known as SARS-CoV-2, has rapidly spread worldwide. Among its many symptoms, ageusia (loss of taste) and anosmia (loss of smell) are considered as hallmarks of infection [1]. Possible mechanisms for loss of smell and taste in the course of COVID-19 disease are being explored [2, 3] but no direct role of the Renin-Angiotensin-aldosterone system (RAAS) in anosmia and ageusia has been suggested until now. Here we raise a mechanistic hypothesis of unexplored functions of RAAS-associated peptidases in modulating senses of taste and smell and its relationship with chemosensory impairment during COVID-19. This mechanistic perspective has implications for personalized medicine and the pharmacogenomics of COVID-19 management.

### RAAS-associated peptidases as cellular coronaviruses receptors in humans

Loss of taste (ageusia) and/or loss of smell (anosmia) have unusually emerged as the most specific symptoms in COVID-19 pandemic outbreak, often being the first or the lone differentiating clinical manifestation of infection by SARS-CoV-2 [1, 4]. During evolution, many coronaviruses have coopted components of the Renin-Angiotensin-Aldosterone System (RAAS) as viral entry receptors [5]. SARS-CoV, the virus responsible for the global outbreak of SARS in 2003, and SARS-CoV-2, the viral etiology of the current COVID-19 pandemic, both use angiotensin-converting enzyme 2 (ACE2) as functional receptor for viral entry [6, 7]. Other coronaviruses such as hCoV-229E, which causes common cold, enter cells via aminopeptidase N (APN or CD13) ref. [8]. ACE2 and APN are both enzymes from RAAS, potentially pointing to this system as a direct contributor for infection and disease progression of coronaviruses in mammals.

**Fig. 1:**
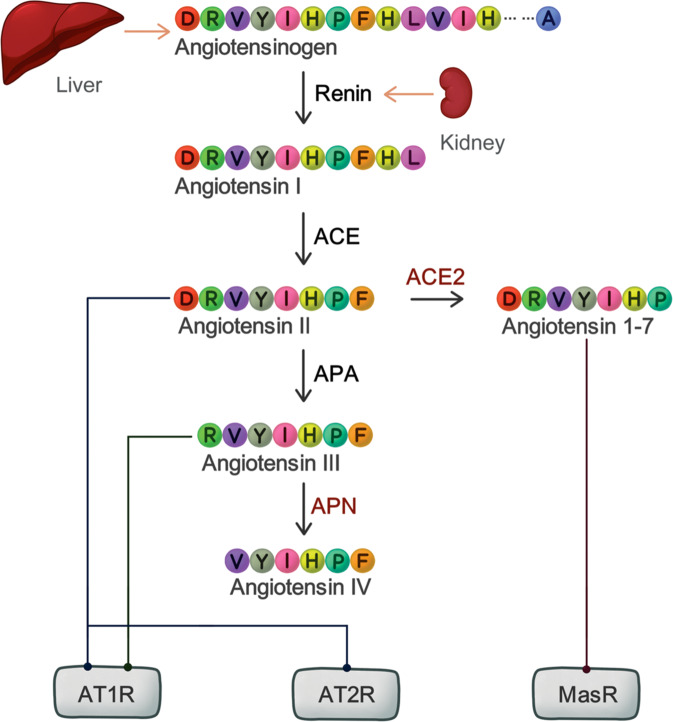
The canonical RAAS pathway. Simplified schematic representation of the RAAS. Light arrows represent secretion, dark arrows represent enzymatic reactions and lines represents bound to receptor. ACE: Angiotensin II Converting Enzyme, APA: aminopeptidase A, APN: aminopeptidase N, AT1R: Angiotensin II type 1 receptor, AT2R: Angiotensin II type 2 receptor, MasR: Mas receptor.

The RAAS (Fig. 1) is best known as an endocrine network that regulates arterial blood pressure and fluid balance homeostasis. It acts systemically through angiotensinogen-derived peptides in blood vessels of the heart and kidney. In brief, liver-produced angiotensinogen is converted into the peptide angiotensin-I (AngI) by the peptidase renin, produced by the kidney. AngI is then cleaved by ACE into AngII, which is a potent vasoconstrictor peptide. However, AngII has a truly short life as it is further converted by aminopeptidase A (APA) and aminopeptidase N (APN) into other metabolite peptides with different bioactivities. Besides these well-documented vascular effects, the RAAS is also known to participate in other complex biological phenomena, such as glucose metabolism, kidney homeostasis, and cancer-related angiogenesis [9–11]. ACE2 and APN, the cell-entry receptors for coronaviruses, are in fact membrane-bound peptidases responsible for processing of RAAS-related peptides, indicating a plausible involvement of this system in related symptoms of infection. Although these proteases have been found in lung, kidney, intestine, and other organs, their expression pattern in sensory tissues in correlation to virus infection is currently under evaluation to determine its potential effects in either oral or nasal epithelium and its role in taste and/or smell impairment during the pathogenesis of viral infection.

### Taste impairment mediated by SARS-CoV-2 infection: Is there a role for the RAAS?

RAAS components are expressed in taste buds of mice and have been shown to modulate perception of salty and sweet flavors [12, 13]. In fact, ACE2 is found in human epithelial cells of the tongue, and its expression is downregulated by SARS-CoV and SARS-CoV-2 infection [14, 15]. Although less frequently reported, oral chemosensory alterations are also present in hCoV-229E infection via APN receptor, suggesting a role of RAAS dysfunction in viral infection-related ageusia and dysgeusia [16]. Thus, we postulate that RAAS might be involved in loss of taste reported during coronaviruses infection course.

**Fig. 2:**
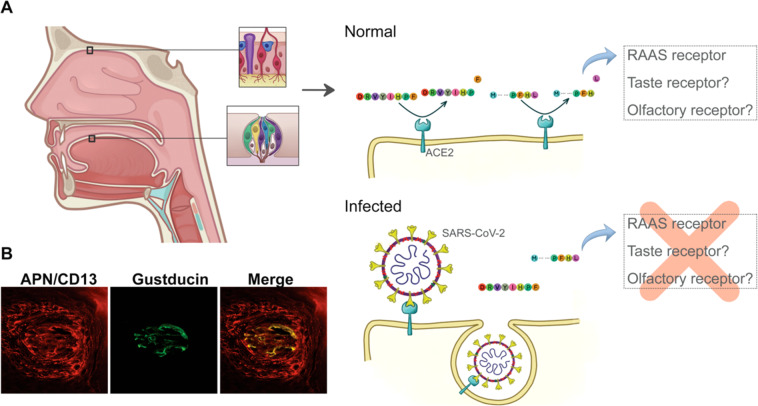
Local RAAS impairment leads to loss of taste and smell during COVID-19. **A**. Hypothesis schematic representation: Local RAAS in nasal and oral tissue drives loss of taste and smell after SARS-CoV-2 infection due to reduced protease activity. **B** Colocalization of APN (CD13) and gustducin. Confocal microscopy image of tongue shows co-localization of APN and gustducin.

Peptidases regulate metabolism of amino acids and flavor perception in food by releasing specific residues [17]. Distinct amino acids have specific flavors such as glutamate, well known for its umami taste and widespread used in food industry as a flavor additive (under the trade name “AJI-NO-MOTO” or “味の素”, “essence of taste”), and some L-amino acids with aromatic side chains, which trigger bitter taste [18, 19]. ACE2 and APN are, respectively, amino- and carboxypeptidases that promote proteolytic cleavage of proteins and peptides. These RAAS proteases expressed in tongue epithelium may promote activation of taste receptors by releasing residues and thus contributing to taste perception. Once coronaviruses binds to ACE2, they are internalized together into cells, reducing ACE2 availability in the cell membrane [20]. Therefore, ageusia and dysgeusia could perhaps reflect insufficient RAAS peptidase function at membrane due to receptor internalization by coronaviruses infection on taste buds (Fig. 2A). Thus, we reasoned that the proteolytic release of amino acids by RAAS peptidases might be an as yet unappreciated component of taste perception.

Some organs have a functional RAAS pathway, which works cooperatively with systemic RAAS to maintenance of body homeostasis [21]. For instance, local RAAS were reported for kidney, working as a paracrine control of salt absorption in glomerulus, whose damage contribute to the pathophysiology of hypertension and renal injury [22]. This also seems to be the case for flavor-perceiving tissues because all components of the pathway are expressed in taste buds, namely renin, angiotensinogen, angiotensin II type-1 receptor (AT1R), and ACE2 [13]. Notably, exogenous AngII suppresses salt and enhance sweet taste responses in mice through AT1R while pharmacological inhibition of AT1R suppresses the AngII effects on taste cells. This mechanism thereby contributes to reduced intake of salt and sugar, providing evidence of RAAS impact on taste sensitivity and food consumption [12]. Furthermore, a large study of self-reported chemosensory dysfunction during COVID-19 from Global Consortium for Chemosensory Research (GCCR) has recently highlighted salty and sweet flavor perception disturbances among the most reported taste-related impairments in SARS-CoV-2 infection, followed by the others [23]. Altogether, these data corroborate the existence of local RAAS pathway in flavor-perceiving tissues and their possible implication in the genesis of COVID-19 infection-related symptoms.

Sweet and salty taste cells are central for energy intake and electrolytic homeostasis, respectively, and it is reasonable that it might have a rapid and regionalized intake control, managed by a local RAAS. Given that ACE2 receptors are expressed in other cell types, as umami, bitter and sour taste receptors, RAAS may also have unexplored roles in these other taste sensors. Likewise, APN is also expressed in mice tongue, co-localizing with G-α-gustducin (Fig. 2B), a G-protein associated with taste and gustatory transduction [24]. As coronaviruses hijack the mammalian cell machinery, other molecules involved in taste processing and signal transduction may be impaired in infected cells, leading to the ageusia and dysgeusia as the presenting and hallmark symptom reported by many COVID-19 patients.

As a tantalizing final piece of evidence supporting the involvement of the RAAS in sensory disturbances, either loss or impairment of taste have been self-reported by some patients during use of angiotensin II receptor blockers or ACE inhibitors, in a dose-dependent manner [25–27]. One should note that, in usual clinical settings, those anti-hypertensive drugs are often under the molar concentrations required for effective peptidase inhibition in oral epithelial and in taste cells, which would explain this peculiar side-effect being reported only by some and not by most or all hypertensive patients receiving RAAS-modulating drugs.

### Sniffing out COVID-19: “A rose by any other name would smell as sweet”

Far less is currently known about the role of the RAAS pathway in olfaction but RAAS enzymes are also expressed in murine and human olfactory epithelium [28, 29]. In both species, ACE2 was found in sustentacular cells, primarily associated with structural support, and in stem and perivascular cells [30]. In contrast, olfactory neurons do not seem to express the entry receptor for SARS-CoV-2 [30, 31]. Although a local RAAS has not as yet been described for olfactory epithelium, the presence of ACE2 in sustentacular cells would arguably favor of a mechanistic role of this system in the processing of odorant molecules. Enzymatic pre-processing of odorants did occur prior to olfactory recognition, as demonstrated to odorants with functional groups (such as aldehydes and esters) that are typically targeted by metabolic enzymes present in mucus. Post-processed metabolites stimulate other odorant receptors and modulate olfactory transmission [32]. Likewise, APN is also expressed and proteolytic active in nasal epithelium [33], directing us to the hypothesis that protease activity of ACE2 and/or APN might be relevant to chemosensory recognition by participating in odorant processing that precedes receptor recognition.

Moreover, sustentacular cells have structural and protective roles on olfactory neurons similar to glial cells in central nervous system. They are tentatively responsible for damage prevention from hazardous molecules to neuronal olfactory cells. In fact, many odorants bound to odorant-binding proteins (OBP) in nasal mucus and are internalized by sustentacular cells, which mediates rapid clearance of molecules from olfactory neurons contact surface [34]. Intercellular communications between sustentacular and neuronal cells also regulate odor perception and their impairment could lead to sense disturbances [35, 36]. The infection of sustentacular cells by SARS-CoV-2 in animal model results in loss of cilia in olfactory sensory neurons [31], what likely impairs processing and internalization of odorants as well as intercommunication between these two cell types. Lastly, reduction in odorant-OBP complexes uptake by infected nasal support cells could also trigger odorant receptors desensitization [37], ultimately resulting in anosmia observed in COVID-19 infected patients.

### SARS-CoV-2 pharmacogenomics and RAAS-related polymorphisms

Is there a role for genetic background in modulating RAAS in the setting of SARS-CoV-2 infections? If so, how is it connected to COVID-19 symptoms? Although sociodemographic data, age and comorbidities correlate with severity of disease, they are not sufficient to explain the high variability in clinical manifestation, outcomes and mortality rates of SARS-CoV-2 infection. These observations have raised an increasing interest in the role that certain host’s genetic backgrounds might play in this scenario [38]. In genomic association studies, two loci were related to morbidity of COVID-19 disease: the *ABO* gene locus, in chromosome 9, and the locus 3p21.31. The *ABO* gene locus was previously known as a source of pharmacogenomic variants correlated to ACE activity; most strikingly, now the exact same genetic locus has been implicated in increased respiratory failure of COVID-19 patients [39, 40]. Regarding polymorphisms in *ACE2* gene itself, it is known that variants in this genetic locus promote pharmacogenetic modulation of ACE inhibitors response [41] and therefore they have now been under scrutiny as genetic susceptibility loci for COVID-19 outcomes [42]. One should note that the *ACE2* locus is located at chromosome Xp22.2 and that, as an X-linked gene, its genetic polymorphisms will have dominant effects in men and may thereby contribute to clarify the increased prevalence of severe COVID-19 cases in male patients. As X-chromosome inactivations in women are mostly random, they might perhaps exert a protective role in target tissues from most severe forms of COVID-19. The new locus implicated in respiratory failure of new coronaviruses disease, located in the chromosome 3, comprises a Neanderthal-inherited region of human genome and was actually shown to be less frequent in the East Asia population [43]. Consistently, there is also a relevant ethnic difference in the prevalence of taste and smell disturbances as symptoms of COVID-19, which are clinically three times less common in East Asia [44]. Thus, one could speculate that this variance results from the differential genetic prevalence of this specific chromosomal region in 3p21, although such theory still remains to be proven. Lastly, the viral genome may be crucial for infection consequences, since the D614G variant of SARS-CoV-2 (the predominant strain in Europe) predisposes infected individuals to suffer from anosmia [45]. In this sense, genomic variants affecting RAAS gene expression or activity likely influences taste and smell sense impairment, although this hypothesis has not as yet been clinically confirmed. Thus, genetic background is relevant to personalized response to SARS-CoV-2 infections and treatment, and could be responsible for disparities observed in COVID-19 drug-repurposing investigational clinical trials [46, 47]. In sum, these data highlights the urgent need for detailed populational pharmacogenomic studies.

### Taste, smell in the setting of COVID-19: the current state of the medical science and knowledge gaps

It is widely accepted that olfactory deficiency influences taste perception through damage in the retronasal olfactory contribution to flavor perception, but one could speculate that both likely occur under a common viral etiology. Data from GCCR report on oral and nasal sensory disturbances sought to minimize olfaction influence in taste report and actually suggested a degree of independence between these factors [23]. Consistently with a transient infective agent, COVID-19-related symptoms seems to last only few weeks, while only long-lasting olfactory disturbances were shown to have influence on taste sense [48].

Self-report of olfaction and gustatory disturbances facilitates populational data acquisition as shown by a smartphone application to symptoms report of non-hospitalized COVID-19-positive individuals [49]. Despite of the increased reach of internet-based platforms or population survey questionnaires, subjectivity of self-reporting may always lead to underestimation of symptoms prevalence [23]. As a matter of fact, when objective olfactory tests were applied, 98% of patients presented some degree of smell dysfunction against only ~60–80% reported by other subjective approaches [1, 50, 51]. Therefore, the application of objective tests to properly evaluate the extent of ageusia and anosmia in patients with suspected or confirmed SARS-CoV-2 infection is clearly desirable.

The nasal and oral epithelia are the main gateways for respiratory virus infections. As receptors for coronaviruses such as ACE2 and APN are expressed in these sensory tissues, one could surmise that these pathogens indeed gain entry through certain nasal and oral cells and control the intrinsic mammalian cell machinery to produce viral particles, thereby altering the native physiology of chemosensory perception. This logical possibility is evidenced by high viral loads found in nasal cavity--which indicates site-specific viral replication in the anatomical site--that is further confirmed by the use of nasal swabs analysis as diagnostic tools [52]. Even if one cannot entirely rule out the contribution of local inflammation to sensory disturbances, unlike other viral infections, COVID-19 loss of taste and smell is rarely reported along with nasal congestion with no significant alterations in paranasal sinuses by computed tomography [23, 53], directing us to other causes than immunologic alterations in sensitive organs by inflammation.

Animal models for coronaviruses infection have been particularly challenging, since infection in mice does not reliably mimic the clinical features often seen in humans. The emergence of transgenic mice expressing human (h)ACE2 and/or hAPN under species-specific promoters [54–56] may allow an improved understanding of viral pathogenesis. Golden Syrian hamsters were recently used as a trustworthy model to olfactory alterations, resembling partial recovery of olfactory epithelium in 14 days post-infection seen in patients [31]. The establishment of non-human primate animal models for SARS-CoV-2 infection and disease are in progress. These models can resemble respiratory failure and mucosal viral loads found in humans [57–59] and may be useful tools to explore smell and taste impairment, perhaps even more reliable than hACE2 transgenic mice. Considering limitations of each species in experimental design, all may be used to unravel the contribution of RAAS to chemosensory disturbances during coronaviral infection, perhaps in combination with classic food-aversion experimental models.

### Final remarks

Several lines of evidence point to RAAS impairment as a central element in the pathophysiology of anosmia and ageusia during initial presentation of SARS-CoV-2 infection in humans. The juxtaposition of a loco-regional functional RAAS system, with SARS-CoV-2 entry receptor expression in sensory organs, and the side-effects of RAAS inhibitors affecting gustatory senses present an interesting research paradigm to unveil the contribution of this ancient system to the pathogenesis of ageusia and anosmia caused by COVID-19 infection. A well-codified symptom-infection relationship will expedite management and isolation of infected people, particularly in developing countries lacking enough specific test capacity. While most patients seem to fully recover from respiratory COVID-19 infections, it remains too early to tell whether there are permanent effects on the precious human senses of taste and smell. Moreover, The COVID-19 Host Genetics Initiative [60], a joint effort that aim to determine genetic variants involved in response to infection and development of disease, will open a promising path to pharmacogenomic exploration of treatment and symptoms management of SARS-CoV-2 infection. Considering the central influence of RAAS in the pathophysiology of COVID-19 infection, research focused on organ-specific consequences may be helpful to understand the disease course and hopefully lead to insights into the development of effective therapies and vaccines.
